# A Tumor in Disguise: Gallbladder Tumor Presenting as Hepatic Abscesses

**DOI:** 10.7759/cureus.44369

**Published:** 2023-08-30

**Authors:** Muhammad Haseeb ul Rasool, Utsow Saha, Arshia K Sethi, Muhammad Adnan, Nahian Rabby

**Affiliations:** 1 Medicine, Icahn School of Medicine at Mount Sinai, Queens Hospital Center, New York City, USA; 2 Internal Medicine, Icahn School of Medicine at Mount Sinai, Queens Hospital Center, New York City, USA; 3 Internal Medicine, Stony Brook University, New York City, USA

**Keywords:** gallbladder mass, gallbladder, hepatobiliary cancers, pyogenic hepatic abscess, hepatic abscess

## Abstract

Gallbladder tumors are the most common tumors of the biliary tract. They are rare but clinically aggressive tumors found either as metastatic disease or occasionally detected upon the histopathological assessment of cholecystectomy biopsy. Adenocarcinoma is the most common phenotype of gallbladder cancer, which can be mild to moderately differentiated. Other malignant phenotypes include mucinous adenocarcinoma, signet cell, small cell, papillary adenocarcinoma, intestinal type adenocarcinoma, and undifferentiated carcinoma. The rarity of the disease makes the diagnosis extremely difficult in the initial phases. Liver abscesses are extremely rare and scarcely reported presentation of gallbladder cancer, with only a handful reported cases. It is speculated that the development of hepatic abscesses depicts direct involvement of hepatic parenchyma, development of associated necrosis, and superimposed bacterial infection evolving to an abscess. Gallbladder perforations are rare and potentially life-threatening complications of any gallbladder disease. Increased intraluminal pressure leads to mural necrosis, emphysematous changes in the wall, and vascular compromise which leads to gallbladder wall necrosis leading to perforation. Gallbladder tumors are exceedingly notorious for poor outcomes with very limited survival.

Here, we present a case of a 69-year-old male who initially presented with impending perforation of the gallbladder with multiple hepatic masses, which were thought to be metastatic deposits. However, on biopsy, he was found to have multiple hepatic abscesses due to localized necrosis. Further workup revealed that the patient had an advanced metastatic gallbladder tumor that had passed the stage of tumor resection. Gallbladder perforations are classified according to Niemeier's classification. Our patient had a type II perforation which resulted in a hepatic abscess.

## Introduction

Gallbladder cancers are the leading malignancy of the biliary system and carry a grave prognosis. Unfortunately, gallbladder cancers usually present with non-specific symptoms that result in delayed diagnosis, resulting in diagnosis at advanced stages when clinical intervention is not very useful. Only one-third of patients are diagnosed at operable stages, most of them being incidental findings in cholecystectomy surgeries [1]. Adenocarcinoma, the most common phenotype of gallbladder cancer, can be mild to moderately differentiated. Other malignant phenotypes include mucinous adenocarcinoma, signet cell, small cell, papillary adenocarcinoma, intestinal type adenocarcinoma, and undifferentiated carcinoma [2]. Gallbladder tumors have been associated with the presence of chronic cholecystitis, and gallstones. Other pathologies associated with an increased risk of gallbladder malignancy include chronic bacterial infections, ascending cholangitis, primary sclerosing cholangitis, gallbladder polyps, anomalous anatomy of the pancreaticobiliary duct, and presence of various cysts [3]. The presence of transluminal calcification is an additional risk factor for the development of gallbladder malignancy. Commonly named as porcelain gallbladder, generalized calcifications are associated with a 62% increased risk of developing gallbladder malignancy, for which prophylactic cholecystectomy is advised [4]. Gallbladder malignancies can spread to the liver either by direct invasion, hematogenous, or lymphatic invasion. In rare cases, rapidly expanding metastasis in the hepatic parenchyma results in necrosis of hepatic parenchymal cells leading to necrosis that appears as abscesses in clinical radiology. Most of the time, these are sterile abscesses but, in a few cases, these can become infected [5].

Here, we present a case of a patient who presented with impending gallbladder perforation with hepatic masses, which was initially thought to be an infective process, but later on, the underlying malignancy was revealed on biopsy.

## Case presentation

**Figure 1:**
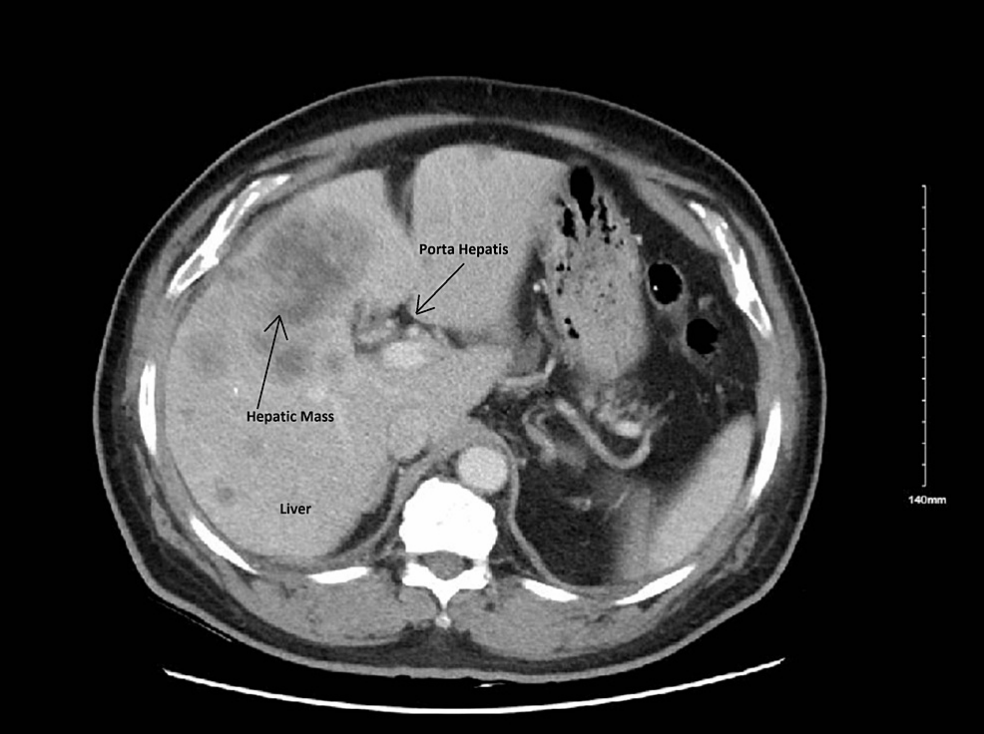
CT abdomen revealing multiple hepatic masses.

**Figure 2:**
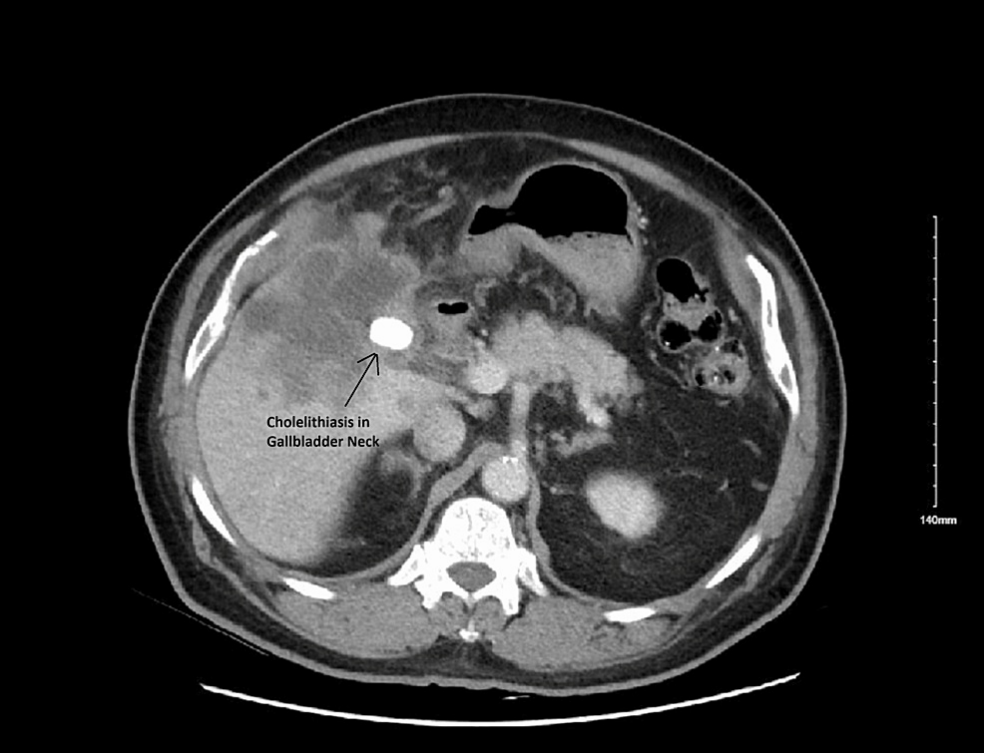
CT abdomen revealing cholelithiasis in the gallbladder neck along with hepatic mass.

A 69-year-old male with a past medical history of diabetes mellitus, hypertension, hyperlipidemia, cerebrovascular disease without residual weakness, cervical radiculopathy, and benign prostatic hyperplasia, presented to the hospital with complaints of new onset generalized constant abdominal pain more in the epigastrium and right upper quadrant area for one week. The pain was not associated with nausea or vomiting but the patient endorsed some weakness and malaise. On examination, the patient was anicteric, the abdomen was mildly distended and tender on superficial palpation globally but more so in the epigastric area. Murphy’s sign was positive. A computerized tomography (CT) scan of the abdomen with intravenous contrast was performed, which revealed numerous lesions in the liver suggestive of liver metastasis, with a 2.3×1.6 cm gallbladder neck stone. The gallbladder neck was irregular and thickened. Fluid in the gallbladder lumen appeared contiguous with an ill-defined low-attenuation mass in the liver parenchyma adjacent to the gallbladder fossa. In the right upper quadrant, below the liver and adjacent to the gallbladder fundus, fat stranding, and free fluid were observed. A partially thickened ring-enhancing rim suggested a developing abscess or biloma. These findings were suggestive of acute cholecystitis possibly with neoplastic involvement of the gallbladder and impending gallbladder-free wall perforation including perforation into the adjacent liver with multiple liver masses (Figures 1, 2).

**Figure 3:**
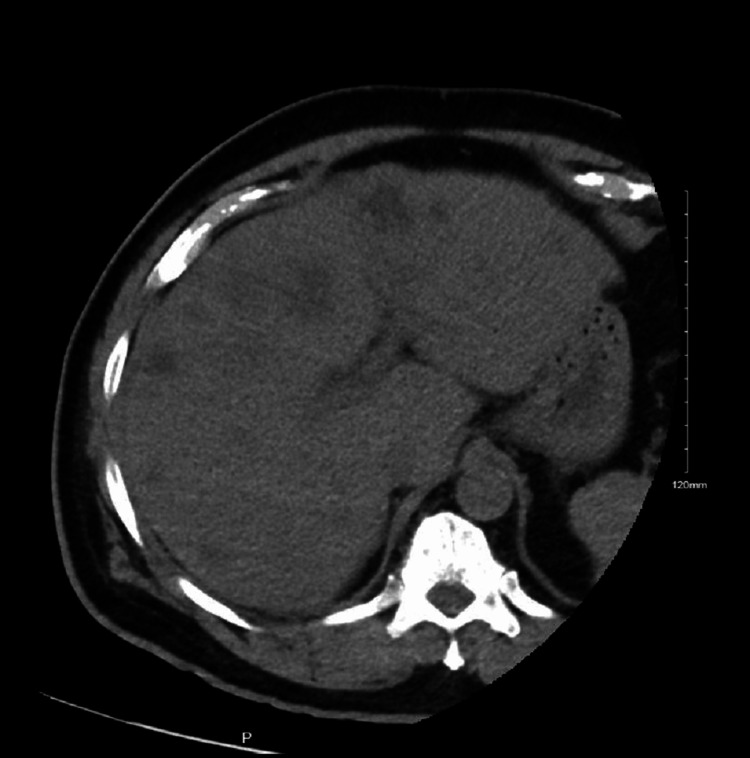
Preoperative images focused on the hepatic area during CT-guided biopsy demonstrated a significant decrease in the size of hepatic masses.

The patient was admitted for intravenous antibiotics and resuscitation. Interventional radiology was consulted for a biopsy of the hepatic lesion. However, during the biopsy of lesions, frank purulent fluid was drained which resulted in a significant decrease in the size of masses (Figure 3).

The purulent fluid, drained from multiple sites in the right hepatic lobe, was sent for culture and sensitivity testing which yielded *Escherichia coli* 48 hours later. Antibiotic treatment with piperacillin-tazobactam combination was started empirically, which was later continued as per the sensitivity results. General surgery was also consulted for assessment for resection of hepatic masses. However, based on the comorbid conditions, hemodynamic status of the patients, and metastasis in bilateral hepatic lobes, he was deemed an unsuitable candidate for resection. Oncology was consulted for further recommendations for treatment. It was recommended to get a biopsy of either the gallbladder or hepatic lesions for tissue diagnosis via biopsy prior to any further intervention. CA 19-9 was 54132 U/mL, with no hyperbilirubinemia and mildly elevated alkaline phosphatase to 716 U/L. On the patient's own request, he was sent home with instructions to follow-up with the oncology clinic once his hemodynamics had normalized after receiving antibiotic treatment. CT-guided hepatic biopsy showed adenocarcinoma with extensive necrosis of uncertain primary site; however, morphologically and immunohistochemically suggestive of gallbladder origin. A colonoscopy was done to rule out synchronous colorectal carcinoma which was negative. Cytokeratin 17 and 20 were positive which was also suggestive of gallbladder cancer. The patient was later admitted again within a month due to hyperkalemia of 6.7 mEq/L with new-onset acute kidney injury (AKI) with a known history of benign prostatic hyperplasia (BPH). The hepatic profile was suggestive of worsening transaminitis with the obstructive pattern. The patient had a distended abdomen on examination. Repeating CT abdomen and pelvis without contrast ruled out any active hepatic abscess and was negative for common bile duct dilation or obstruction. Magnetic resonance cholangiopancreatography (MRCP) was done to rule out any hepatic abscess but gallstones and liver metastasis with ascites were noted. The patient then developed symptoms of small bowel obstruction. He refused the nasogastric tube, and general surgery was consulted. In their opinion, it was secondary to advancing malignancy and no surgical intervention was performed. He was treated with sennosides and lactulose, resulting in partial improvement in obstructive symptoms.

Nephrology was consulted for AKI and it was suggested that AKI was either due to hepatorenal syndrome or abdominal compartment syndrome. Volume expansion with colloid was recommended. Renal function improved a little with albumin supplementation but gradually worsened. The CA 19-9 level was 54000U/L and the CEA level was 561 ng/mL. During this visit, the patient denied any fever/septic symptoms. The patient had persistent elevated WBC in the range of 20s, but it was thought to be due to malignancy. Hematology and oncology were consulted again, and they deferred chemotherapy at this time due to the poor prognosis of the diseases and also due to liver and renal dysfunction. Considering the patient's functional status and apparent prognosis, the family opted to transition the patient to comfort care. Unfortunately, the patient expired shortly after the transition was made.

## Discussion

Although gallbladder (GB) carcinoma is the most common carcinoma of the biliary tree, its low incidence, about four in 10 of every cancer of the biliary tree, makes it a rare encounter for clinicians [6]. GB carcinoma, similar to cholecystitis has a higher female incidence and a higher age preponderance. It also shows a higher predilection in people from Chile, Poland, India, Japan, and Israel than in other parts of the world [7]. GB carcinoma can be of various types with the most common being adenocarcinoma, and less likely squamous or adenosquamous carcinoma. Among all, mucinous adenocarcinoma is seen in approximately 2.5% of cases [8].

The most common presentation is an incidental finding, in the setting of a known benign cause such as gallstones or during cholecystectomy for the same. Certain cases aren’t detected until pathology reverts back after gallbladder removal with that being the most common mode of presentation making it customary to evaluate surgically resected tissue [9]. Other methods of presentation can include constitutive symptoms like malaise, weight loss, or more specific features like right upper quadrant pain. Patients can also present with signs and symptoms of obstructive jaundice, likely signifying a more progressive disease course [7].

GB cancer presenting as a hepatic abscess is one of the most uncommon presentations for the tumor with only a few cases reported in the literature so far [10]. Rich hepatic blood supply from the both hepatic artery and portal vein predisposes to both hepatic metastases of tumor and the spread of pyogenic bacteria from the gut causing hepatic abscess [11]. The hepatic abscess can be a common presentation in the case of a gallstone, usually caused by pressure necrosis resulting in gallbladder perforation. This perforation is classified using the Neimer classification to aid management [12]. Our patient had a type II perforation which resulted in a hepatic abscess. In cases of abscess along with metastasis, the presentation may be atypical, with signs pointing both to an infectious and a malignant etiology. The presence of bacteria in the abscess can further complicate diagnosis, by pointing to a primary colonic origin of the cancer. Therefore, a needle biopsy becomes essential to establish a diagnosis [13].

Most gallbladder tumors present at a late stage as they metastasize early. Modes of metastasis include direct, lymphatic, vascular, neural, intraperitoneal, and intraductal [11]. Although abdominal ultrasound is the first test to be performed, diagnosis is usually made by histopathological examination. Contrast-enhanced CT is another superior modality not only for diagnosis but also for assessing tumor extent and invasion [14]. MRI is another non-invasive way that is now essential for diagnosing hepatic abscesses and metastasis [14]. Although tumor markers like CEA and CA 19-9 can be elevated, they lack diagnostic significance because of low sensitivity and specificity [15]. Nevertheless, cytokeratin 17 and CD 10 positivity is associated with poor tumor differentiation and a higher chance of distant metastasis whereas MUC6 positivity showed a favorable prognosis in well to moderately-differentiated tumors [16].

The primary method of treatment is surgical resection but it is usually limited by patient factors and stage at presentation [17]. The likelihood of detection at a later stage makes it unresectable, which unfortunately is the case in almost 75% of patients, resulting in poor prognosis [7]. Lack of submucosa in the gallbladder and serosa in the liver enables the easy hepatic progression of any GB disease and thus results in a dismal prognosis [18].

In our case, the patient presented with multiple hepatic metastases complicated with abscesses positive for *E. coli*. Although the abscesses were drained and the colonoscopy was negative for any primary tumor, surgical resection was not considered due to extensive metastasis and the poor condition of the patient. The patient rapidly deteriorated to death within a few months of presentation.

## Conclusions

Gallbladder malignancies are one of the most common malignancies of the biliary tree. Most of the patients are diagnosed at advanced stages when surgical resection is not possible. In advanced malignancies with multiple hepatic metastases, rapidly growing malignancies can result in localized necrosis due to compromised blood supplies. Such lesions present as hepatic abscesses, upon drainage, most of these are sterile but can get infected due to ascending infections. It is important to keep gallbladder malignancies on the list of differential diagnoses for patients with risk factors for gallbladder tumors and for patients in whom the source of infection in abscesses cannot be clinically demonstrated.
